# Purinergic Signalling in Snakebite Envenomation: Mechanistic Insights, Therapeutic Targets and Future Perspectives

**DOI:** 10.3390/molecules31203609

**Published:** 2026-10-10

**Authors:** Dhébora Albuquerque Dias, Maria Helena Souza Soares, Rita de Cássia Avellaneda Guimarães

**Affiliations:** 1 Laboratory of Food Physicochemical Analysis (LFQA), Faculty of Pharmaceutical Sciences, Food and Nutrition (FACFAN), Federal University of Mato Grosso do Sul (UFMS), Av. Costa e Silva s/n, Cidade Universitária, Campo Grande 79070-900, MS, Brazil; 2 Undergraduate Program in Food Engineering, Faculty of Pharmaceutical Sciences, Food and Nutrition (FACFAN), Federal University of Mato Grosso do Sul (UFMS), Av. Costa e Silva s/n, Cidade Universitária, Campo Grande 79070-900, MS, Brazil; m.helena@ufms.br; 3 Graduate Program in Health and Development in the Central-West Region of Brazil, Medical School, Federal University of Mato Grosso do Sul (UFMS), Campo Grande 79070-900, MS, Brazil; rita.guimaraes@ufms.br

**Keywords:** snakebite envenomation, purinergic signalling, ATP, adenosine, P2X7 receptor, ectonucleotidases

## Abstract

Snakebite envenomation is a major neglected tropical disease associated with substantial morbidity and mortality. Its pathophysiology involves complex interactions between venom components and host responses, leading to inflammation, tissue damage, and systemic alterations. Purinergic signalling has emerged as an important mechanistic pathway underlying these processes. This narrative review was based on a comprehensive literature search of PubMed, Scopus, and Web of Science, focusing on purinergic signalling, snake venoms, inflammasome activation, extracellular nucleotide metabolism, and adjunct therapeutic strategies. Extracellular ATP, acting as a damage-associated molecular pattern (DAMP), activates purinergic receptors and amplifies inflammatory cascades, vascular dysfunction, and cell death pathways. CD39 and CD73 regulate extracellular nucleotide metabolism and generate adenosine, which exerts immunomodulatory and vascular effects. Venom-derived nucleotide-hydrolysing enzymes may also directly modulate host purinergic signalling, supporting an integrated network of host- and venom-derived mechanisms. Purinergic signalling represents a promising mechanistic target for adjunct therapy in snakebite envenomation; however, the temporal dynamics, efficacy, and safety of purinergic-targeted interventions require further preclinical and clinical validation.

## 1. Introduction

Snakebite envenomation remains a major global public health challenge, particularly in tropical and subtropical regions, where it disproportionately affects rural and socioeconomically vulnerable populations. An estimated 5.4 million snakebites occur worldwide each year, resulting in substantial mortality and long-term disability, and the condition is recognized as a neglected tropical disease [1,2,3]. In Brazil, the majority of envenomation cases are caused by species of the genus *Bothrops*, while other medically important genera include *Crotalus*, *Lachesis*, and *Micrurus* [4].

Although antivenom therapy remains the cornerstone of treatment, it has limited efficacy in preventing local tissue injury, including pain, edema, hemorrhage, and necrosis, which frequently persist despite effective control of systemic manifestations [5,6]. These limitations highlight the need for adjunct therapeutic strategies targeting the early inflammatory and cellular events that contribute to local tissue damage following envenomation [7,8,9].

Snake venoms are complex mixtures of enzymatic and non-enzymatic components whose composition varies according to species, geographic distribution, age, and environmental factors, contributing to marked differences in toxicity and clinical presentation [10]. These venoms contain proteins, peptides, and small molecules capable of modulating multiple biological systems, including hemostatic, neuromuscular, and inflammatory pathways [3,11]. Notably, several venom components have been shown to influence extracellular nucleotide metabolism and purinergic signalling, including apyrases, CD73-like ectonucleotidases, and CD39-like phosphodiesterases identified in different snake species [12,13,14,15].

At the tissue level, venom-induced cytotoxicity promotes the release of intracellular constituents into the extracellular milieu, including extracellular nucleotides, particularly ATP, and nucleosides that act as signalling molecules. These mediators participate in the purinergic signalling system, a regulatory network composed of extracellular purines and pyrimidines, ectonucleotidases, and cell surface receptors of the P1 and P2 families [16,17]. Beyond its classical role in neurotransmission, purinergic signalling is now recognized as a key regulator of inflammation, immune cell activation, vascular responses, platelet function, nociception, and regulated cell death [18,19].

Increasing evidence indicates that snake venoms not only trigger host purinergic responses indirectly through tissue damage but may also directly modulate purinergic signalling through venom-derived nucleotidases, phosphodiesterases, bioactive nucleosides, and extracellular vesicles carrying purinergic-related components [20,21]. Venom-derived nucleotidases, phosphodiesterases, and related enzymatic activities have been identified in several snake species, suggesting that these components directly contribute to extracellular nucleotide metabolism [12,13,14,15].

In this context, an experimental study using *Bothrops mattogrossensis* venom demonstrated modulation of specific receptor and ectonucleotidase components of the purinergic system, specifically altered expression of the P2X7 receptor (P2X7R) and the ectonucleotidases CD39 and CD73, accompanied by changes in inflammatory and oxidative stress markers [22]. These findings support the hypothesis that purinergic signalling contributes to the pathophysiology of snake venom-induced tissue injury [22].

Given its well-established role in inflammasome activation, inflammation, and regulated cell death, P2X7R has emerged as a promising pharmacological target. However, its specific contribution to the pathophysiology of snakebite envenomation remains to be fully elucidated [19,23,24].

Taken together, these findings support the view that purinergic signalling is closely involved in the pathophysiology of snakebite envenomation through the dynamic interplay between venom-derived purinergic components and host purinergic responses [12,13,14,15,22]. However, the mechanistic interplay between venom-derived purinergic components and host purinergic signalling networks remains incompletely understood, limiting the identification of novel therapeutic targets and the development of mechanism-based adjunct therapies.

This review integrates current evidence on purinergic signalling in snakebite envenomation to provide a mechanistic framework describing the dynamic interplay between host- and venom-derived purinergic pathways, thereby advancing our understanding of envenomation pathophysiology and identifying potential targets for mechanism-based adjunct therapeutic strategies.

## 2. Literature Search Strategy

This narrative review was based on a comprehensive literature search conducted using PubMed, Scopus, and Web of Science. The search was performed using combinations of terms related to purinergic signalling and snakebite envenomation/envenoming, including “snake venom ATP”, “purinergic signalling in envenomation”, “purinergic signalling in snakebite envenoming”, “purinergic receptor in envenomation”, “purinergic receptors in snakebite envenoming”, “ATP in envenomation”, “ATP in snakebite envenoming”, “purinergic system in snakebite envenomation”, “purinergic system in snakebite envenoming”, “ectonucleotidases in snake venom”, “nucleotidases in snake venom”, “CD39 in snake venom”, “CD73 in snake venom”, “adenosine in snake envenomation”, “adenosine in snakebite envenoming”, “extracellular nucleotides in snake envenomation”, “extracellular nucleotides in snakebite envenoming”, “nucleotide release in snake envenomation”, “P2X7 receptor in snake envenomation”, “P2X7 receptor in snakebite envenoming”, “NLRP3 inflammasome in snake envenomation”, “NLRP3 inflammasome in snakebite envenoming”, “purinergic signalling inflammation snake venom”, “purinergic signalling inflammation snakebite envenoming”, “purinergic signalling snake venom treatment”, “purinergic signalling treatment snakebite envenoming”, “purinergic receptors therapeutic targets snakebite”, and “purinergic receptors therapeutic targets snakebite envenoming”.

The searches identified 536 records (Scopus, *n* = 326; Web of Science, *n* = 196; PubMed, *n* = 14). After the removal of 178 duplicate records, 358 unique records remained for title and abstract screening. Of these, 288 records were excluded, and 70 publications were retained for detailed eligibility assessment.

Publications were considered relevant when they provided evidence addressing at least one of the following: (i) purinergic receptors or purinergic signalling in snakebite envenomation or experimental models involving snake venom or venom toxins; (ii) snake venom enzymes involved in extracellular nucleotide or nucleoside metabolism, including 5′-nucleotidases, phosphodiesterases, apyrases/ADPases, and related ectonucleotidase-like activities; (iii) ATP, ADP, AMP, adenosine, or other purines/pyrimidines investigated in the context of snake venom actions; or (iv) functional consequences of these pathways relevant to envenomation, including inflammation, inflammasome activation, pain, platelet aggregation, coagulation, vascular responses, and tissue injury. Original research articles, review articles, and the relevant clinical literature published primarily in English were considered, with seminal studies retained when necessary to provide historical or mechanistic context.

Studies were excluded when purinergic-related terms were incidental to the study question; snake venom enzymes were used solely as analytical or biochemical reagents; studies were restricted to enzyme purification, assay development, or catalytic/structural characterization without a biologically relevant functional connection to envenomation; nucleotide-related experiments were unrelated to snake venom or envenomation; or venomic/proteomic studies merely reported the presence of nucleotide-metabolizing enzymes without providing relevant biological or mechanistic information. Biochemical characterization studies were not excluded solely because they involved enzyme purification when they also demonstrated a biologically relevant functional effect, such as nucleotide hydrolysis associated with inhibition of platelet aggregation or anticoagulant activity.

Relevance was assessed according to the experimental question and biological contribution of each publication rather than solely by the occurrence of search terms. Evidence obtained directly from snake venoms, isolated venom components, or experimental envenomation models was distinguished from the broader purinergic literature used to provide mechanistic context.

During peer review, additional publications were identified and incorporated to address specific mechanistic points raised by the reviewers. These additional studies were assessed according to the same relevance and eligibility criteria described above.

## 3. Purinergic System

The purinergic system comprises an extracellular signalling network that coordinates the actions of nucleotides (e.g., ATP and ADP) and nucleosides (e.g., adenosine) through specific receptors and ectonucleotidases, thereby regulating inflammation, vascular responses, nociception, and cell death [18,23,25]. Purinergic receptors are classified into two families: P1 receptors, which are adenosine-activated G protein-coupled receptors, and P2 receptors, which are further subdivided into P2X ligand-gated ion channels and P2Y G protein-coupled receptors activated by extracellular nucleotides such as ATP and ADP [18,26].

Extracellular ATP and related nucleotides are rapidly metabolised by a coordinated network of ectonucleotidases. E-NTPDases, including CD39, sequentially hydrolyse ATP to ADP and AMP, whereas ecto-nucleotide pyrophosphatase/phosphodiesterases (E-NPPs), particularly ENPP1, can hydrolyse ATP directly to AMP with concomitant generation of inorganic pyrophosphate (PPi). Ecto-5′-nucleotidase (CD73) subsequently converts AMP to adenosine, thereby regulating receptor activation and the duration of purinergic signalling [27,28,29,30]. This enzymatic cascade governs the balance between pro-inflammatory ATP-driven signalling and adenosine-mediated immunoregulatory effects [27,28,29,31].

This regulatory axis is particularly relevant in conditions associated with extensive tissue damage, such as snakebite envenomation. In this context, venom-induced cytotoxicity promotes the release of intracellular ATP into the extracellular space, where it acts as a damage-associated molecular pattern (DAMP), activating purinergic receptors and amplifying local inflammatory responses [19,31].

The P2X7R is one of the best-characterized purinergic receptors involved in inflammatory signalling owing to its ability to trigger nucleotide-binding oligomerization domain (NOD)-, leucine-rich repeat (LRR)- and pyrin domain-containing protein 3 (NLRP3) inflammasome activation and IL-1β release in response to elevated extracellular ATP [32,33,34]. In addition, P2Y receptors contribute to leukocyte recruitment and vascular responses [35], whereas adenosine generated by ectonucleotidase activity modulates immune responses and tissue repair through activation of P1 receptors, particularly the A2AR and A2BR receptor subtypes [31,36].

Among P2Y receptors, ADP, UTP, and UDP activate distinct receptor subtypes and intracellular signalling pathways. ADP primarily activates P2Y1, P2Y12, and P2Y13 receptors. P2Y1 is predominantly coupled to Gq/11 proteins, leading to phospholipase C activation and intracellular Ca^2+^ mobilization, whereas P2Y12 and P2Y13 are mainly coupled to Gi/o proteins and inhibit adenylyl cyclase, thereby reducing intracellular cAMP levels [37]. In platelets, P2Y1 and P2Y12 cooperate in ADP-mediated activation and aggregation. UTP primarily activates P2Y2 and P2Y4 receptors, whereas UDP preferentially activates P2Y6; these receptors predominantly signal through Gq/11-dependent phospholipase C activation and Ca^2+^ mobilization [37]. Through their effects on platelets, immune cells, and vascular cells, these nucleotide-specific pathways may contribute to haemostatic, inflammatory, immune, and vascular responses during envenomation. However, the direct contribution of individual P2Y receptor subtypes to snakebite pathophysiology remains insufficiently characterized, and these proposed connections are therefore based largely on established purinergic mechanisms rather than direct evidence from snakebite models. Nevertheless, direct venom evidence supports the capacity of snake venom nucleotidases to alter extracellular nucleotide availability: a 5′-nucleotidase isolated from *Trimeresurus gramineus* (common bamboo pit viper) venom hydrolysed ADP, promoted adenosine formation, and inhibited ADP-induced platelet aggregation [38].

Collectively, these mechanisms suggest that the purinergic system may provide an integrative mechanistic link between venom-induced tissue damage and downstream inflammatory, vascular, and nociceptive responses associated with snakebite envenomation [18,19].

This framework establishes a mechanistic basis for understanding how host-derived and venom-derived purinergic components may interact during snakebite envenomation and supports the rationale for exploring purinergic pathways as potential targets for adjunct therapeutic strategies [23,24].

## 4. Purinergic Components in Venoms

Snake venoms comprise complex mixtures of enzymatic and non-enzymatic components that extend beyond the major toxin families to include molecules capable of modulating extracellular nucleotide metabolism and, consequently, purinergic signalling. In addition to well-characterised toxins such as phospholipases A_2_ (PLA_2_) and metalloproteinases, several venom constituents exhibit nucleotide-hydrolysing activities, thereby modulating purine (ATP, ADP) and pyrimidine (UTP, UDP) signalling pathways within the purinergic system [15,39,40].

Extracellular purine and pyrimidine nucleotides function as important signalling molecules that activate purinergic receptors, whereas ectonucleotidases regulate the signalling cascade by sequentially hydrolysing these molecules into downstream metabolites, thereby controlling the magnitude and duration of purinergic signalling [27,28,29].

Early biochemical studies demonstrated that snake venoms possess ATPase and ADPase activities capable of degrading extracellular nucleotides [41,42], providing a biochemical basis for potential effects on nucleotide-dependent haemostatic and inflammatory processes. This evidence has since been broadened by comparative and functional studies across different snake taxa. Sales and Santoro detected ATPase, ADPase, 5′-nucleotidase, and phosphodiesterase activities in a panel of Brazilian snake venoms, with particularly prominent nucleotide-hydrolysing activities in representatives of *Bothrops*, *Crotalus*, and *Lachesis* [39]. Functional experiments further demonstrated that 5′-nucleotidase activity contributes to the anticoagulant effect of *Naja naja* (Indian cobra) venom [43], while the purified nucleotide pyrophosphatase/phosphodiesterase NPP-BJ from *Bothrops jararaca* hydrolysed extracellular nucleotides and inhibited ADP-induced platelet aggregation [44]. Similarly, CdcPDE isolated from *Crotalus durissus collilineatus* (South American rattlesnake) inhibited ADP-induced platelet aggregation in a dose-dependent manner and was cytotoxic to human keratinocytes [45]. Additional functional studies further broaden this evidence across viperid genera: phosphodiesterases purified from *Vipera lebetina*, *Daboia russelli russelli*, and *Trimeresurus stejnegeri* hydrolysed ADP and inhibited platelet aggregation, supporting a conserved capacity of venom PDEs to modulate ADP-dependent haemostatic responses [46,47,48]. Direct chemical analyses have also identified biologically relevant concentrations of adenosine in *Bitis arietans* (puff adder), *Bitis gabonica* (Gaboon viper), and *Bitis nasicornis* (rhinoceros viper) venoms [49,50]. Together, these studies provide direct experimental evidence that venom-derived nucleotide-metabolising enzymes and purine metabolites can alter extracellular nucleotide availability and haemostatic responses; broader receptor-specific inflammatory consequences remain mechanistic interpretations that require direct validation in envenomation models.

More recent evidence has provided mechanistic insight into how venom-derived components interact with host purinergic signalling. Proteomic and functional analyses of *Bothrops mattogrossensis* (boca-de-sapo) venom demonstrated ectonucleotidase-like activities together with modulation of CD39, CD73, and P2X7R expression, accompanied by alterations in inflammatory and oxidative stress markers in both in vitro and in vivo models [22]. These findings suggest that venom-derived enzymes not only metabolise extracellular nucleotides but may also modulate the purinergic microenvironment within envenomed tissues. Importantly, the *B. mattogrossensis* findings constitute direct evidence from venom-based experimental models, whereas the extension of these observations to specific receptor-driven mechanisms across other snake species should be regarded as a mechanistic hypothesis rather than a universally demonstrated effect.

An additional layer of complexity is introduced by venom-associated extracellular vesicles (EVs). Proteomic analyses revealed that extracellular vesicles isolated from *Bothrops jararaca* venom are enriched in 5′-nucleotidase (CD73-like) and other venom-related proteins and are capable of interacting with mammalian cells [20]. Earlier experimental work on *Gloydius blomhoffi* also demonstrated that ecto-5′-nucleotidase can be associated with exosome-like vesicles in fresh venom, providing independent evidence that vesicular carriage of nucleotide-metabolising enzymes is not restricted to *Bothrops* [51]. More recently, extracellular vesicles derived from *Naja naja* and *Daboia russelii* venoms were shown to exhibit ATPase and ADPase activities, suggesting a vesicle-mediated mechanism for the delivery of nucleotide-modulating enzymes into host tissues [21]. These observations are consistent with experimental evidence demonstrating ectonucleotidase-like activities in *Bothrops mattogrossensis* venom, accompanied by modulation of host purinergic components during envenomation [22]. Together, these findings suggest that venom-associated extracellular vesicles and soluble venom enzymes may cooperatively contribute to extracellular nucleotide turnover and modulation of purinergic signalling within the envenomed tissue microenvironment. Collectively, these observations support a model in which venom-derived components directly influence extracellular nucleotide availability through the enzymatic degradation of extracellular nucleotides and the delivery of nucleotide-modulating enzymes and nucleosides [21,22,42].

In parallel, host-derived ATP can be rapidly released from venom-damaged cells, where it acts as an endogenous DAMP capable of activating purinergic receptors and amplifying inflammatory signalling [19,32,52]. This interplay between venom-derived and host-derived purinergic components establishes a dynamic regulatory network that shapes inflammation, vascular dysfunction, and tissue injury during snakebite envenomation.

Importantly, direct evidence of rapid ATP release after venom-induced tissue injury, together with venom-associated nucleotide metabolism, supports the possibility that purinergic signalling is engaged early in the pathological process [19,22,52]. These temporal dynamics provide a mechanistic basis for investigating timely pharmacological modulation of purinergic pathways as a potential adjunct therapeutic strategy to attenuate venom-induced inflammation and tissue damage.

These findings support the notion that purinergic signalling may represent an integrative mechanism in snakebite pathophysiology by linking venom-derived and host-derived purinergic processes. This integrated perspective provides a mechanistic basis for investigating purinergic pathways as potential targets for mechanism-based adjunct therapeutic strategies and offers new opportunities to advance our understanding of envenomation pathophysiology and guide the development of innovative therapeutic interventions [22,23,24]. The direct venom evidence summarized above—including comparative nucleotidase profiling, purified venom phosphodiesterases with functional effects on ADP-dependent platelet responses, and direct detection of venom adenosine—supports the biochemical plausibility of this framework across multiple genera [39,43,44,45,49].

The main purinergic components identified in snake venoms, together with their reported activities and proposed biological relevance, are summarized in Table 1.

## 5. Integrated Mechanistic Model of Purinergic Signalling in Snakebite Envenoming

Purinergic signalling plays a central and dynamic role in the pathophysiology of snakebite envenomation, acting both as a mediator of tissue damage and as a modulator of immune and vascular responses. Snake venom components induce rapid cellular injury, leading to the extracellular release of ATP, UTP, and other nucleotides from damaged cells. Experimental studies have demonstrated that venom-induced myonecrosis is accompanied by rapid ATP release into the extracellular space, where it functions as a DAMP, initiating inflammatory signalling [52]. These extracellular nucleotides activate P2X and P2Y receptors expressed on endothelial, immune, and resident tissue cells, thereby triggering downstream inflammatory signalling cascades [18,23].

In parallel, venom-associated enzymes, including phosphodiesterases and other nucleotide-hydrolysing enzymes, may further influence extracellular nucleotide availability through the hydrolysis of ATP and related nucleotides, thereby modulating receptor activation [15]. Consistent with this concept, proteomic and functional analyses of *B. mattogrossensis* venom identified purinergic system components, providing additional evidence that venom-derived purinergic molecules contribute to the regulation of host inflammatory responses during envenomation [22].

Among purinergic receptors, P2X7R is a key regulator of inflammatory signalling owing to its ability to activate the NLRP3 inflammasome and amplify inflammatory responses [23]. Activation of P2X7R induces cation fluxes, including Ca^2+^ influx and K^+^ efflux, and stimulates the generation of reactive oxygen species (ROS), partly through NADPH oxidase activation and mitochondrial dysfunction [54]. These events promote NLRP3 inflammasome assembly, leading to caspase-1 activation and the subsequent maturation and release of IL-1β and IL-18 [34,54,55]. Recent evidence further supports the role of P2X7R as a promising pharmacological target in inflammatory disorders [24]. Although direct evidence in snakebite envenoming remains limited, these findings provide a mechanistic basis for investigating P2X7R as a potential target for adjunct therapeutic strategies.

Extracellular nucleotides are hydrolysed by the membrane-bound ectonucleotidases CD39 and CD73, generating adenosine and potentially shifting the local signalling environment from ATP-driven pro-inflammatory signalling toward adenosine-mediated immunoregulation [25]. Recent experimental evidence further demonstrated that CD73-mediated extracellular adenosine production constitutes an important mechanism for immune regulation under inflammatory conditions [56]. Adenosine subsequently activates P1 receptors, particularly A2AR and A2BR, thereby exerting anti-inflammatory, immunomodulatory, and tissue-protective effects through the suppression of pro-inflammatory signalling pathways [32]. Consequently, this enzymatic cascade dynamically regulates the magnitude and duration of the inflammatory response, facilitating its resolution while promoting tissue protection and restoration of homeostasis [57].

This dual mechanism, involving pro-inflammatory signalling mediated by P2 receptors and anti-inflammatory signalling mediated by P1 receptors, establishes a dynamic and temporally regulated purinergic network at the site of envenomation. During the early phase of envenomation, extracellular ATP released from damaged tissues may act as an important signalling molecule, promoting inflammation, immune cell activation, and tissue injury through activation of P2 receptors. As ATP and other extracellular nucleotides are hydrolysed by CD39 and CD73, adenosine-mediated immunoregulatory signalling may become increasingly relevant [25]. Consequently, adenosine activates P1 receptors, particularly A2AR and A2BR, promoting inflammatory resolution, tissue protection, and restoration of tissue homeostasis through suppression of pro-inflammatory signalling pathways, including NF-κB [32,57].

Snake venoms contain several purinergic components, including extracellular nucleotides, nucleosides, phosphodiesterases, and ectonucleotidase-like enzymes, which may modulate extracellular purine metabolism and receptor activation [15,42]. Experimental studies have identified phosphodiesterases and ectonucleotidases in snake venoms, including CD39-like phosphodiesterases and CD73-like ectonucleotidases, which modulate extracellular nucleotide metabolism and may contribute to the regulation of haemostasis and inflammation [13,14,15,58].

Extracellular vesicles derived from *B. jararaca* venom have been shown to carry purinergic-associated proteins and interact with mammalian cells, providing additional evidence that venom-derived components participate in host–venom communication [20]. Likewise, proteomic and functional analyses of *B. mattogrossensis* venom demonstrated the presence of purinergic system components, further supporting this concept [22]. These findings also suggest that interspecific variation in the abundance and composition of venom-derived purinergic components may influence local tissue injury and inflammatory responses among different snake species, although this hypothesis requires further comparative investigation [3].

In addition to its established role in inflammation, purinergic signalling may also contribute to the neurobiological manifestations of snakebite envenoming. Activation of P2X7R and other purinergic receptors expressed on peripheral neurons and glial cells contributes to nociceptive signalling, neuroinflammation, and pain sensitization [23,24]. Experimental studies have shown that pharmacological inhibition of P2X7R attenuates inflammatory and neuropathic pain, highlighting this receptor as a promising, although still underexplored, target for adjunct therapeutic strategies [59].

The integration of venom-derived purinergic components with host purinergic signalling provides a comprehensive mechanistic perspective for understanding the coordinated regulation of inflammation, vascular dysfunction, tissue injury, and subsequent reparative responses during snakebite envenomation. Recent experimental evidence demonstrating the presence of purinergic system components in *B. mattogrossensis* venom further reinforces the notion that venom-derived purinergic molecules contribute to the interplay between venom components and host purinergic signalling [22]. Overall, the available evidence identifies purinergic signalling as a key integrative axis linking venom activity to host responses and provides a mechanistic basis for investigating purinergic pathways as potential targets for mechanism-based adjunct therapeutic strategies [23,24].

The rapid kinetics of ATP release, Ca^2+^ influx, ROS generation, and extracellular nucleotide hydrolysis support the view that purinergic signalling is engaged during the early stages of snakebite envenomation. These temporal dynamics provide a mechanistic basis for investigating the early, temporally targeted modulation of purinergic pathways as a potential adjunct therapeutic strategy, particularly through selective modulation of P2X7R and adenosine receptor signalling [19,32].

## 6. Purinergic Signalling in Inflammation

Snakebite envenomation induces rapid tissue injury, leading to the immediate extracellular release of ATP and other nucleotides at the bite site [52]. This early accumulation of extracellular ATP can initiate purinergic signalling by acting as a DAMP and activating P2 receptors, including P2X7R, expressed on immune and resident tissue cells [18,23]. Subsequent activation of P2X7R promotes NLRP3 inflammasome assembly, caspase-1 activation, and the maturation and release of the pro-inflammatory cytokines IL-1β and IL-18 [54,55].

Within this inflammatory microenvironment, host-derived ATP release coincides with venom-derived enzymatic activities that modulate extracellular nucleotide availability [15,22]. Venom components, including nucleotide-hydrolysing enzymes and extracellular vesicle-associated ectonucleotidase activities, regulate purine metabolism and thereby shape the local purinergic microenvironment [15,20,21]. Depending on venom composition and the stage of envenomation, these mechanisms may either amplify inflammatory signalling or facilitate its resolution through dynamic regulation of extracellular purinergic signalling [22].

Importantly, the relatively low sensitivity of P2X7R to ATP does not necessarily conflict with the presence of venom-derived nucleotide-hydrolysing enzymes. Venom-induced membrane damage can generate rapid and spatially restricted increases in extracellular ATP near injured cells, creating transient ATP-rich microenvironments before substantial nucleotide degradation occurs. Experimental studies with *Bothrops* myotoxins support this spatiotemporal interpretation: *Bothrops asper* (terciopelo) myotoxins induce a rapid and large ATP efflux from damaged skeletal muscle [52], while Bothropstoxin-I from *Bothrops jararacussu* induces marked ATP release from myotubes and promotes ATP/P2X7R-dependent K^+^ efflux, NLRP3 inflammasome activation, and IL-1β production [60]. Conversely, in vivo bioluminescence studies with *B. asper* showed that purified myotoxins induce rapid ATP release, whereas complete venom produces little detectable ATP because venom nucleotidases efficiently convert ATP into ADP, AMP, and adenosine [61]. Thus, ATP release and nucleotide hydrolysis are not mutually exclusive processes but are temporally and spatially coupled: transient high local ATP concentrations may permit early P2X7R activation, whereas subsequent hydrolysis limits the magnitude and duration of ATP-driven signalling and progressively shifts the extracellular milieu toward adenosine. However, ATP concentrations within receptor-proximal microdomains have not yet been directly quantified in envenomed tissues, and this spatiotemporal model therefore requires further experimental validation.

Extracellular nucleotides are hydrolysed by the ectonucleotidases CD39 and CD73, generating adenosine and potentially increasing the contribution of adenosine-mediated immunoregulatory signalling [25]. Adenosine subsequently activates P1 receptors, particularly A2AR and A2BR, thereby exerting anti-inflammatory, immunomodulatory, and tissue-protective effects through suppression of pro-inflammatory signalling pathways [32]. Consequently, this enzymatic cascade limits the magnitude and duration of the inflammatory response, facilitating inflammatory resolution while promoting tissue protection and the restoration of homeostasis [57].

The temporal dynamics of purinergic signalling are likely to be a critical determinant of disease progression during snakebite envenomation. The rapid release of extracellular nucleotides, followed by their sequential enzymatic metabolism, provides a mechanistic basis for investigating early pharmacological modulation of purinergic pathways as a potential adjunct therapeutic strategy to limit excessive inflammation and tissue injury [19,32]. However, the optimal timing, duration, and receptor specificity of such interventions remain to be established in the context of snakebite envenomation. Delayed intervention may reduce the therapeutic efficacy of strategies aimed at limiting inflammasome activation and the downstream inflammatory cascade.

Purinergic signalling also contributes to vascular dysfunction and leukocyte recruitment, both of which are hallmarks of snakebite envenomation [3]. Activation of P2X and P2Y receptors regulates endothelial permeability, platelet activation, and immune cell migration under inflammatory conditions [23]. These mechanisms establish a mechanistic link between purinergic signalling and vascular leakage, oedema, haemorrhage, and other inflammatory manifestations associated with snakebite envenomation [8,25].

Pain is another clinically important manifestation in which P2X receptor subtypes beyond P2X7R may participate. Direct experimental evidence is available for P2X2- and P2X3-containing receptors. BomoTx, a Lys49 myotoxin from *Bothrops moojeni* (Brazilian lancehead) venom, induces ATP release from sensory neurons through a mechanism involving pannexin hemichannels, and the released ATP subsequently activates P2X2 and/or P2X3 receptors. In vivo, BomoTx produces inflammatory pain, thermal hyperalgesia, and mechanical allodynia, with genetic deletion of P2X2 and P2X3 significantly attenuating toxin-induced mechanical hypersensitivity [62]. These findings provide direct evidence that P2X2/P2X3 signalling contributes to venom-induced nociception. By contrast, although P2X4R is well established as a mediator of inflammatory and neuropathic pain in other experimental contexts, direct evidence specifically linking P2X4R to pain during snakebite envenomation has not yet been established. Thus, the contribution of individual P2X receptor subtypes to snakebite-associated pain remains incompletely characterized and warrants further investigation.

The available evidence indicates that purinergic signalling exerts a dual and temporally regulated role in snakebite envenomation, acting both as a driver of inflammation and vascular dysfunction and as a mediator of inflammatory resolution and tissue repair. This integrated model provides a mechanistic basis for investigating purinergic pathways as potential targets for mechanism-based adjunct therapeutic strategies.

## 7. Therapeutic Targets

Snakebite envenomation triggers rapid activation of purinergic signalling through the extracellular release of ATP and other nucleotides at the bite site, initiating inflammatory and tissue-damaging responses. Experimental evidence from inflammation and tissue injury models indicates that purinergic signalling follows a temporally regulated pattern, in which early ATP-driven responses promote inflammation and tissue injury, whereas later adenosine-mediated signalling contributes to inflammatory resolution and tissue repair [19,32]. Although this temporal model has not yet been fully established in snakebite envenomation, it provides a mechanistic basis for understanding disease progression and identifying therapeutic opportunities. Accordingly, pharmacological strategies targeting purinergic signalling should consider not only receptor specificity but also the timing of intervention.

P2X7R antagonists have emerged as attractive pharmacological candidates because of the receptor’s central role in inflammasome activation, IL-1β release, and amplification of inflammatory responses [23]. Experimental studies have shown that pharmacological inhibition of P2X7R attenuates inflammatory responses and tissue damage in preclinical models, supporting its potential as a therapeutic target [24]. Pharmacological inhibition of excessive or sustained P2X7R activation may limit Ca^2+^ influx, ROS generation, and subsequent inflammasome activation, thereby attenuating local tissue injury and amplification of inflammatory cascades during envenomation. However, this therapeutic strategy remains to be directly validated in experimental models of snakebite envenomation, and the optimal timing of P2X7R inhibition has not been established.

Experimental P2X7R antagonists include Brilliant Blue G, a selective P2X7R blocker [63], and A-740003, a selective antagonist with documented antinociceptive and anti-inflammatory activity in preclinical models [59]. These findings, together with the well-established role of the receptor in inflammatory signalling, support further investigation of P2X7R antagonists as potential adjunct therapies for snakebite envenomation [23,24]. However, their efficacy and safety remain to be evaluated in experimental models of snakebite envenomation.

Given the central role of P2X7R in host defence and immune regulation, systemic inhibition of this receptor may also impair physiological immune responses, highlighting the need for carefully controlled, context-specific, and temporally optimized therapeutic strategies [23,24].

Importantly, P2X7R signalling should not be considered exclusively detrimental during the early phase of envenomation. Extracellular ATP released immediately after tissue injury functions as a danger signal that can promote early innate immune activation, and P2X7R-expressing immune effector cells participate in protective responses during acute tissue trauma [64]. Therefore, premature or excessive P2X7R inhibition could potentially interfere with beneficial mechanisms involved in the initial recognition of tissue damage and immune-cell activation. In snakebite envenomation, this issue is particularly relevant because ATP availability changes rapidly as a consequence of both tissue injury and venom-associated nucleotide-hydrolysing activities [52,60,61]. Thus, P2X7R inhibition should not be regarded as uniformly beneficial during the earliest phase of envenomation. Rather, a potential therapeutic window may arise when P2X7R/NLRP3 signalling becomes excessive or sustained and contributes to amplification of local tissue injury. However, the temporal boundary between potentially beneficial early P2X7R signalling and detrimental sustained activation has not yet been experimentally defined in snakebite models. Dedicated time-course studies using selective P2X7R modulation are therefore required before an optimal therapeutic window can be established.

**Figure 1:**
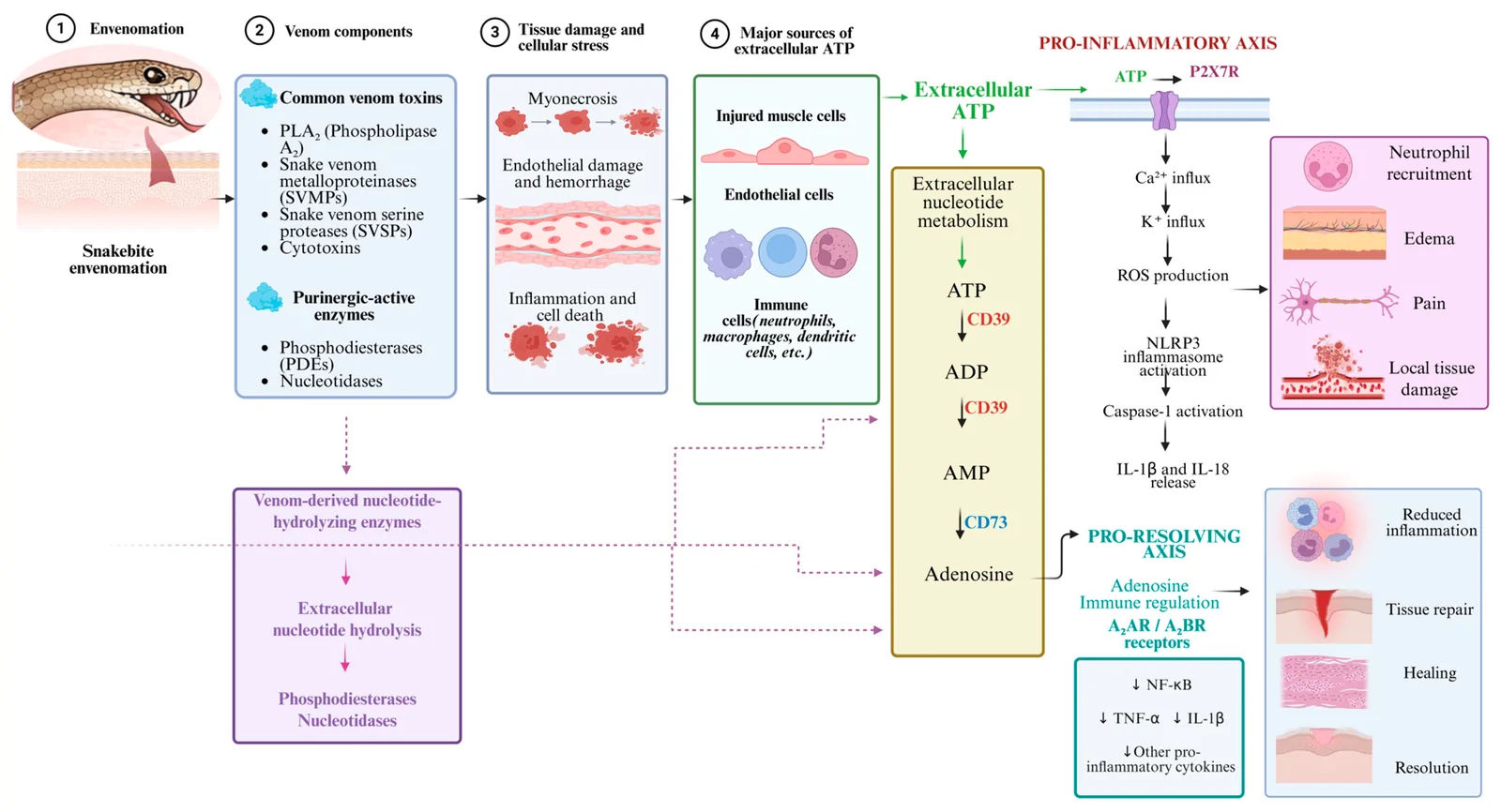
Integrated mechanistic model of purinergic signalling in snakebite envenomation. Snakebite induces tissue damage and cellular stress, leading to the extracellular release of ATP from injured muscle, endothelial, and immune cells. Extracellular ATP activates the P2X7 receptor (P2X7R), promoting Ca^2+^ influx, reactive oxygen species (ROS) production, NLRP3 inflammasome assembly, caspase-1 activation, and the release of the pro-inflammatory cytokines IL-1β and IL-18, ultimately resulting in local inflammation and tissue injury. In parallel, extracellular nucleotides are sequentially hydrolysed by the ectonucleotidases CD39 and CD73, generating adenosine, which activates P1 receptors and promotes immune regulation, tissue protection, repair, and the resolution of inflammation. Venom-derived nucleotide-hydrolysing enzymes, including phosphodiesterases and nucleotidases, may further modulate extracellular nucleotide metabolism, thereby contributing to the regulation of purinergic signalling during envenomation. Solid arrows indicate established or sequential mechanistic relationships, whereas dashed arrows indicate proposed mechanisms that remain to be experimentally validated in snakebite envenomation. Colors are used to visually distinguish the pro-inflammatory and pro-resolving pathways and their associated components. Created in BioRender. Dias, D. (2026). https://BioRender.com/1rgyj5y.

These interconnected pathways highlight the dynamic balance between P2X7R-driven pro-inflammatory responses and adenosine-mediated inflammatory resolution, reinforcing the notion that temporally regulated purinergic signalling represents a potential therapeutic target in snakebite envenomation. Figure 1 summarizes this integrated mechanistic model. Although further experimental validation is required, the available evidence supports continued investigation of purinergic pathways as potential targets for adjunct therapies aimed at limiting tissue injury while preserving physiological immune responses.

P2Y receptors may also represent potential therapeutic targets. These receptors regulate platelet activation and aggregation, leukocyte migration, and vascular tone under inflammatory conditions, suggesting that they may contribute to the inflammatory and haemostatic alterations associated with snakebite envenomation [31,36,65]. However, owing to the diversity of P2Y receptor subtypes and their tissue-specific expression patterns, selective receptor modulation will be essential to maximize therapeutic efficacy while minimizing adverse effects.

Adenosine receptor agonists, particularly those targeting the A2AR and A2BR receptor subtypes, exert anti-inflammatory and tissue-protective effects by limiting neutrophil infiltration and promoting inflammatory resolution [32]. Enhancement of adenosine signalling through receptor agonists or the modulation of ectonucleotidase activity therefore represents a potential strategy for adjunct therapy alongside conventional antivenom treatment, particularly for mitigating local tissue damage [25,57]. However, the therapeutic potential of these approaches remains to be validated in preclinical models of snakebite envenomation.

Evidence from experimental models of inflammatory diseases indicates that activation of A2AR suppresses NF-κB signalling, reduces pro-inflammatory cytokine production, and promotes tissue repair [32,57]. These findings provide a mechanistic basis for investigating the potential role of A2AR activation in promoting inflammatory resolution during snakebite envenomation. In addition, selective A2AR agonists, such as CGS21680, have demonstrated anti-inflammatory and tissue-protective effects in experimental models, supporting their evaluation as adjunct therapeutic agents, although their efficacy in snakebite envenomation remains to be investigated [66]. Importantly, recent preclinical evidence has provided direct support for the therapeutic potential of adenosine itself in snakebite envenomation. Huang et al. demonstrated that early administration of adenosine protected mice against *Bungarus multicinctus* venom-induced lethality in a dose-dependent manner and attenuated venom-induced muscle and membrane damage and neuromuscular blockade [53]. These findings provide direct experimental evidence supporting adenosine as a potential early therapeutic intervention in neurotoxic snakebite envenomation, although the receptor-dependent mechanisms underlying its protective effects and its translational applicability require further investigation.

Ectonucleotidases such as CD39 and CD73 also represent potential therapeutic targets because they regulate the extracellular balance between ATP and adenosine [25]. Recent experimental evidence has further demonstrated that CD73-mediated extracellular adenosine production constitutes an important endogenous mechanism of immune regulation [56]. Accordingly, pharmacological modulation of these enzymes may shift the purinergic microenvironment toward an anti-inflammatory and tissue-protective state by enhancing extracellular adenosine availability [32,57]. Nevertheless, the therapeutic efficacy of this approach is likely to depend on the local tissue microenvironment, the temporal dynamics of extracellular nucleotide metabolism, and the stage of envenomation. Therefore, this strategy still requires validation in preclinical models of snakebite envenomation.

Experimental studies have shown that increased CD39/CD73 activity enhances extracellular ATP degradation and promotes adenosine generation under inflammatory conditions [25]. In the context of snakebite envenomation, modulation of this pathway provides a mechanistic basis for promoting anti-inflammatory signalling and tissue protection. However, excessive or premature modulation may also impair beneficial early immune responses, underscoring the importance of temporally controlled therapeutic intervention [32,57].

The therapeutic window for targeting purinergic signalling is likely to be context- and stage-dependent rather than defined solely by the earliest possible intervention [19,32]. The rapid release of extracellular nucleotides and their subsequent enzymatic metabolism indicate that purinergic signalling changes dynamically after envenomation [52,60,61]. Because early ATP/P2X7R signalling may participate in the initial host response to tissue injury, indiscriminate P2X7R blockade immediately after envenomation could suppress potentially beneficial immune functions [64]. Conversely, excessive or sustained P2X7R/NLRP3 activation may amplify inflammatory tissue damage and therefore represent a more appropriate stage for therapeutic inhibition [23,24,60]. At present, however, the transition between these phases has not been experimentally defined in snakebite models. Accordingly, the timing, duration, and degree of P2X7R modulation should be established in dedicated time-course studies before a sequential purinergic-targeted therapeutic strategy can be proposed.

Although most experimental evidence currently available has been derived from studies of *Bothrops* envenomation, similar purinergic mechanisms may also operate in other medically important genera, including *Crotalus*, *Lachesis*, and *Micrurus*. However, whether these mechanisms are conserved across different snake taxa remains unclear and warrants comparative investigation, particularly in light of the well-established interspecific variability in venom composition [3,10]. These considerations suggest that purinergic-targeted therapies could have broader translational potential, although this possibility remains to be confirmed experimentally. Nevertheless, interspecific differences in venom composition and pathophysiology should be carefully considered when developing adjunct therapeutic strategies.

## 8. Conclusions

Purinergic signalling emerges as a potential integrative mechanism in snakebite envenomation, linking venom-induced tissue injury with the initiation, amplification, and subsequent resolution of inflammatory responses. Early extracellular ATP release functions as a danger-associated signal that activates purinergic receptors, promoting inflammasome activation, cytokine release, oxidative stress, and tissue injury. As extracellular nucleotides are progressively hydrolysed by CD39 and CD73, adenosine-mediated signalling may become increasingly relevant, contributing to inflammatory resolution, tissue protection, and restoration of tissue homeostasis.

This review proposes an integrated mechanistic model in which venom-derived purinergic components and host purinergic signalling dynamically interact throughout envenomation. Rather than representing isolated molecular events, extracellular nucleotide metabolism, purinergic receptor activation, and ectonucleotidase activity constitute a coordinated regulatory network that shapes inflammatory, vascular, immune, and reparative responses. This integrated perspective expands current understanding of snakebite pathophysiology and identifies purinergic signalling as a biologically plausible target for adjunct therapeutic intervention.

Current evidence further suggests that therapeutic modulation of purinergic signalling should consider not only receptor specificity but also the temporal dynamics and biological context of pathway activation. Rather than assuming that immediate P2X7R inhibition is uniformly advantageous, future studies should determine when protective early ATP/P2X7R signalling transitions to excessive or sustained inflammatory signalling and whether subsequent enhancement of adenosine/P1 receptor-mediated responses can promote resolution and tissue repair. These stage-dependent therapeutic concepts remain to be validated in clinically relevant experimental models before translation into clinical practice.

Future studies should define the temporal profile of purinergic signalling during envenomation, characterize these mechanisms across medically important snake genera, and evaluate the efficacy and safety of purinergic-targeted interventions. Integrative approaches combining venom composition, purinergic signalling dynamics, immune responses, and pharmacological modulation will be essential for developing mechanism-based adjunct therapies that complement conventional antivenom treatment and improve the clinical management of snakebite envenomation.

## Figures and Tables

**Table 1:** Purinergic components identified in snake venoms, their reported activities, and proposed biological relevance in snakebite envenomation. The table summarizes purinergic enzymes, purine and pyrimidine nucleosides, extracellular vesicle-associated components, and their reported or proposed biological roles in different snake species and genera. Proposed biological roles are based on direct experimental evidence from snake venoms whenever available or, where appropriate, on mechanistic inferences supported by the broader purinergic signalling literature.

Year	Purinergic Component	Species/Genus	Family	Reported Activity and Proposed Biological Relevance	Reference
2002	Free adenosine (purine nucleoside)	*Naja atra* (Chinese cobra)	Elapidae	High concentrations of free adenosine associated with hypotension and sedative effects in experimental models.	[42]
2002	Free adenosine (purine nucleoside)	*Naja kaouthia*	Elapidae	Cardiovascular and neuromodulatory effects consistent with activation of adenosine receptors.	[42]
2002	Free adenosine (purine nucleoside)	*Bungarus multicinctus*	Elapidae	Acts as a pharmacological co-factor that enhances neurotoxic effects.	[42]
2002	Free purine nucleosides (guanosine, inosine)	*Dendroaspis polylepis*	Elapidae	Indirect modulation of vascular and neural responses attributed to free purine nucleosides.	[42]
2005	Purine and pyrimidine nucleosides	Multiple elapid species	Elapidae	Quantitative HPLC analyses demonstrating the widespread distribution of purine and pyrimidine nucleosides and their potential contribution to hypotensive and neuromodulatory effects.	[50]
2008	ATPase/ADPase activity	Multiple genera	Elapidae/Viperidae	Enzymatic hydrolysis of extracellular ATP and ADP. The potential contribution to extracellular adenosine accumulation and modulation of purinergic signalling is inferred from established purinergic pathways.	[39]
2008	ATPase/ADPase/5′-nucleotidase activity	*Crotalus durissus*	Viperidae	Enzymatic activities involved in extracellular nucleotide hydrolysis, including ATPase, ADPase, and 5′-nucleotidase activities, supporting modulation of extracellular nucleotide availability and purinergic signalling during envenomation.	[39]
2018	Free purine nucleosides and nucleotide-related metabolites	*Micrurus fulvius* (and other coral snakes)	Elapidae	Metabolomic and compositional evidence supporting the presence of purine-related compounds potentially involved in cardiovascular and neuromodulatory effects.	[40]
2018	Free purine nucleosides (low-molecular-weight fraction)	Multiple elapid and viperid species	Elapidae/Viperidae	Metabolomic profiling identified multiple purine nucleosides, supporting their hypothesised contribution to hypotensive and neuromodulatory effects.	[40]
2022	5′-Nucleotidase (extracellular vesicle-associated)	*Bothrops jararaca*	Viperidae	Proteomic evidence demonstrating extracellular vesicles enriched in 5′-nucleotidase and other enzymes potentially involved in extracellular nucleotide metabolism and modulation of purinergic signalling.	[20]
2023	Snake venom phosphodiesterase (svPDE)	Multiple snake species	Elapidae/Viperidae	Structural and evolutionary analyses demonstrate conserved phosphodiesterase activity involved in extracellular nucleotide hydrolysis, supporting a potential role in the modulation of purinergic signalling during envenomation.	[15]
2025	Ectonucleotidase-like activity (CD39/CD73-like)	*Bothrops mattogrossensis*	Viperidae	Proteomic and functional evidence demonstrating CD39/CD73-like ectonucleotidase activity associated with extracellular ATP–adenosine metabolism and modulation of inflammatory and oxidative stress responses.	[22]
2025	ATPase/ADPase activity (extracellular vesicle-associated)	*Naja naja*; *Daboia russelii*	Elapidae/Viperidae	Extracellular vesicle-associated ATPase and ADPase activities contributing to purinergic modulation and extracellular nucleotide turnover during envenomation.	[21]
2026	Adenosine (therapeutic administration)	*Bungarus multicinctus*	Elapidae	Early administration of adenosine protected against venom-induced lethality and attenuated muscle and membrane damage and neuromuscular blockade, supporting its potential as an early therapeutic intervention.	[53]

Abbreviations: ATP, adenosine triphosphate; ADP, adenosine diphosphate; HPLC, high-performance liquid chromatography. Notes: Not all proposed biological roles have been directly demonstrated in snakebite envenomation models; in some cases, they are based on mechanistic interpretations derived from established purinergic signalling pathways. ATPase, ADPase, 5′-nucleotidase, and ectonucleotidase-like activities refer to experimentally detected enzymatic activities and do not necessarily indicate the direct molecular identification of the corresponding enzymes.

## Data Availability

No new data were created or analyzed in this study. Data sharing is not applicable to this article.

## Abbreviations

The following abbreviations are used in this manuscript:

ADP	Adenosine diphosphate
ADPase	Adenosine diphosphatase
AMP	Adenosine monophosphate
ATP	Adenosine triphosphate
ATPase	Adenosine triphosphatase
CD39	Ectonucleoside triphosphate diphosphohydrolase-1 (E-NTPDase1)
CD73	Ecto-5′-nucleotidase (NT5E)
DAMP	Damage-associated molecular pattern
E-NPPs	Ecto-nucleotide pyrophosphatases/phosphodiesterases
E-NTPDases	Ectonucleoside triphosphate diphosphohydrolases
IL	Interleukin
NF-κB	Nuclear factor kappa B
NLRP3	NLR family pyrin domain-containing 3
P2X7R	P2X7 receptor
P2Y	P2Y purinergic receptor
PLA_2_	Phospholipase A_2_
ROS	Reactive oxygen species
SVMPs	Snake venom metalloproteinases
SVPLA_2_	Snake venom phospholipases A_2_
TNF-α	Tumour necrosis factor-alpha
UDP	Uridine diphosphate
